# Disentangling Nature and Nurture in Cognitive Aging Among African American Twins

**DOI:** 10.1093/geroni/igaf122.1296

**Published:** 2025-12-31

**Authors:** Kimson Johnson, Keith Whitfield

**Affiliations:** University of Michigan Institute for Social Research, Ann Arbor, Michigan, United States; University of Nevada, Las Vegas, Las Vegas, Nevada, United States

## Abstract

Higher educational attainment is linked to better cognition in later life, with twin studies helping to disentangle genetic and environmental contributions. Few studies have examined African American twins, particularly in relation to memory performance. This study examines the relationship between education, early-life factors, and cognitive outcomes in older African American twins, focusing on genetic and environmental contributions, stratified by zygosity. Data from the Carolina African American Twins Study of Aging (n = 138, 69 twin pairs) were used to assess cognitive outcomes in adults aged 50 and older, focusing on educational attainment and genetic influences. Correlation analyses examined the relationship between education and cognition (global functioning, working memory, processing speed), stratified by monozygotic (MZ) and dizygotic (DZ) twins. Analyses adjusted for sociodemographic factors, parental education, and childhood health. Overall, findings suggest education-cognition relationships vary slightly across twin types. For global functioning, MZ twins had a slightly higher correlation with education (r = 0.58) than DZ twins (r = 0.55). In fluid reasoning, DZ twins (r = 0.46) had a slightly higher correlation for education compared to MZ twins (r = 0.44). In logical reasoning, the correlation was stronger for MZ twins (r = 0.42) than DZ twins (r = 0.35). Genetics had little impact on the education-cognition relationship. Age showed a small negative correlation with cognition, while parental education had a modest positive impact. Education’s positive impact on cognitive function highlights the importance of improving educational opportunities to support cognitive health in older adults, potentially reducing cognitive aging regardless of genetic predispositions.

